# Supplementary material for: Deep mutational scanning identifies Cas1 and Cas2 variants that enhance type II-A CRISPR-Cas spacer acquisition
Source: Nat Commun. 2025 Jul 1;16:5730. doi: 10.1038/s41467-025-60925-9 (PMC12216489; doi:10.1038/s41467-025-60925-9)
Supplement: Supplementary file 1 — Supplementary Information [file 41467_2025_60925_MOESM1_ESM.pdf]

# Supplementary Information

## Deep mutational scanning identifies Cas1 and Cas2 variants that enhance type II-A CRISPR-Cas spacer acquisition

Raphael Hofmann[1]*, Calvin Herman[1], Charlie Y. Mo[1,2], Jacob Mathai[1] and Luciano A. Marraffini[1,3]*

[1] Laboratory of Bacteriology, The Rockefeller University, New York, NY, USA.

[2] Current address: Department of Bacteriology, University of Wisconsin, Madison, WI, USA.

[3] Howard Hughes Medical Institute, The Rockefeller University, New York, NY, USA

* Correspondence: rhofmann@rockefeller.edu, marraffini@rockefeller.edu

**This PDF file includes:**

Supplementary Figures 1–18

## SUPPLEMENTARY FIGURES

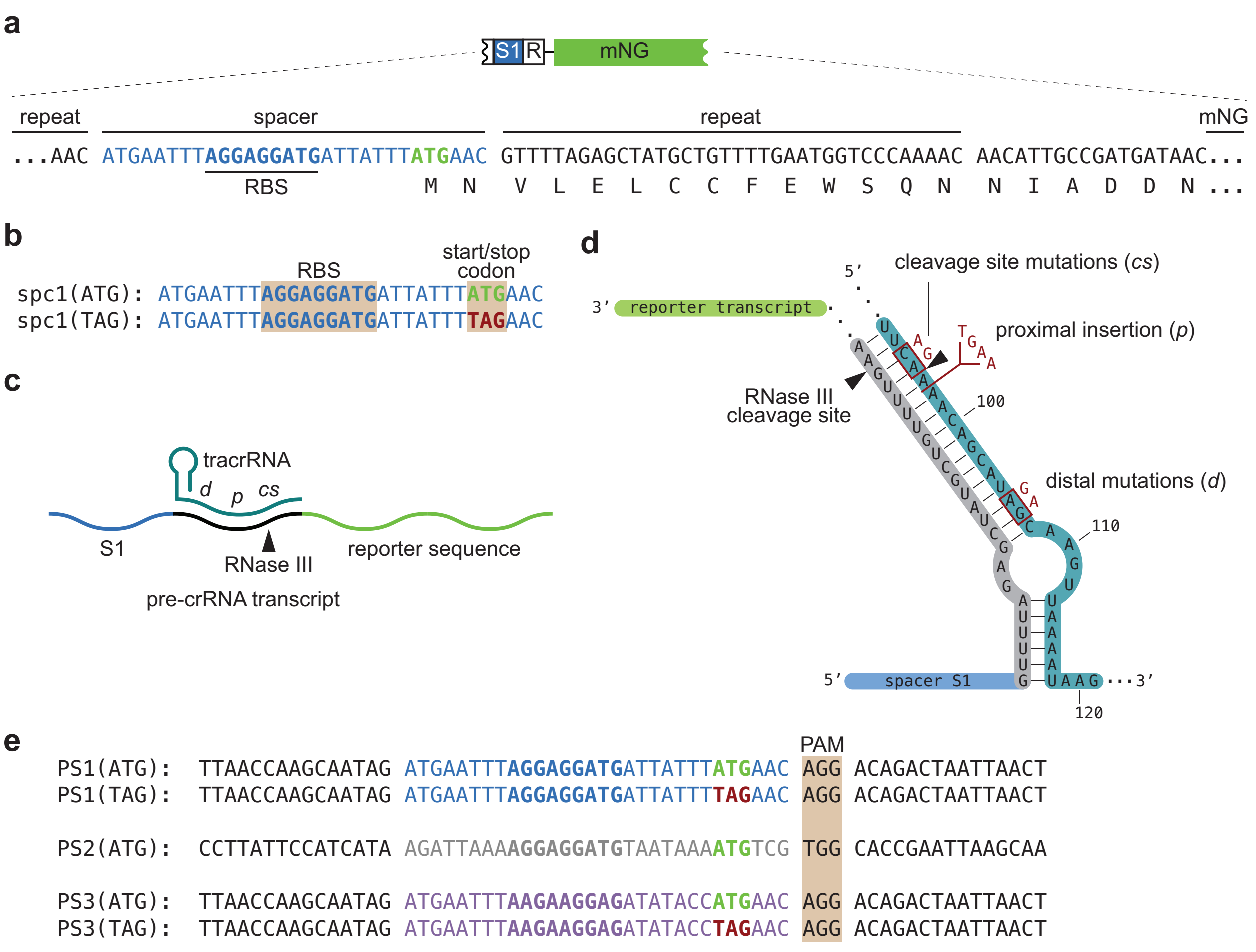


**f**

| Spacer sequence | Spacer length (nt) | Match | PAM |
|---|---|---|---|
| TTTTAGGAGGCAAAA**ATG**GATAAGAAATAC | 30 | plasmid (*cas9* start site) | TCA |
| TTCTTGGGGAGGCTTTTG**ATG**AATCTTAAT | 30 | plasmid (*csn2* start site) | TTT |
| TTAGTCTTGGAGGTTTCAGATT**ATG**AAACAg | 31 | chromosome (*cspA* start site) | GGT |
| ATTATACCAAAGGAGTGATTAT**ATG**TCTCAg | 31 | chromosome (*pepF* start site) | GGT |

**Supplementary Fig. 1 | Development and sequences of reporter strains. a** DNA sequence of the CRISPR array with a spacer containing a translation start site with a ribosome binding side (RBS) and a start codon (ATG), followed by the mNG gene. **b** Sequence of the engineered spacers *spc1(ATG)* and *spc1(TAG)*, with either a start (ATG) or stop (TAG) codon, respectively. **c** Schematic representation of the dsRNA generated by the annealing of the tracrRNA to the repeat sequence (black line) of the pre-crRNA. The location of the mutations shown in **d** are indicated. **d** Mutations introduced in the tracrRNA to suppress crRNA maturation, located in the distal or proximal regions (*d* or *p*, respectively) to the RNase III cleavage site, or at the cleavage site (*cs*). **e** Prespacer sequences used in this study; spacer sequences are shown in blue, grey and purple, and flanking sequences in black. PAM, protospacer-adjacent motif. **f** Sequences of spacers present in a subset of colonies obtained in control experiments after electroporation of PS1(TAG) or a mock electroporation. The putative RBS and ATG are underlined and bolded, respectively. The location of the sequences that match these spacers as well as their associated PAM are indicated. Lowercase letters indicate a mismatch between the spacer and matching sequence.

a

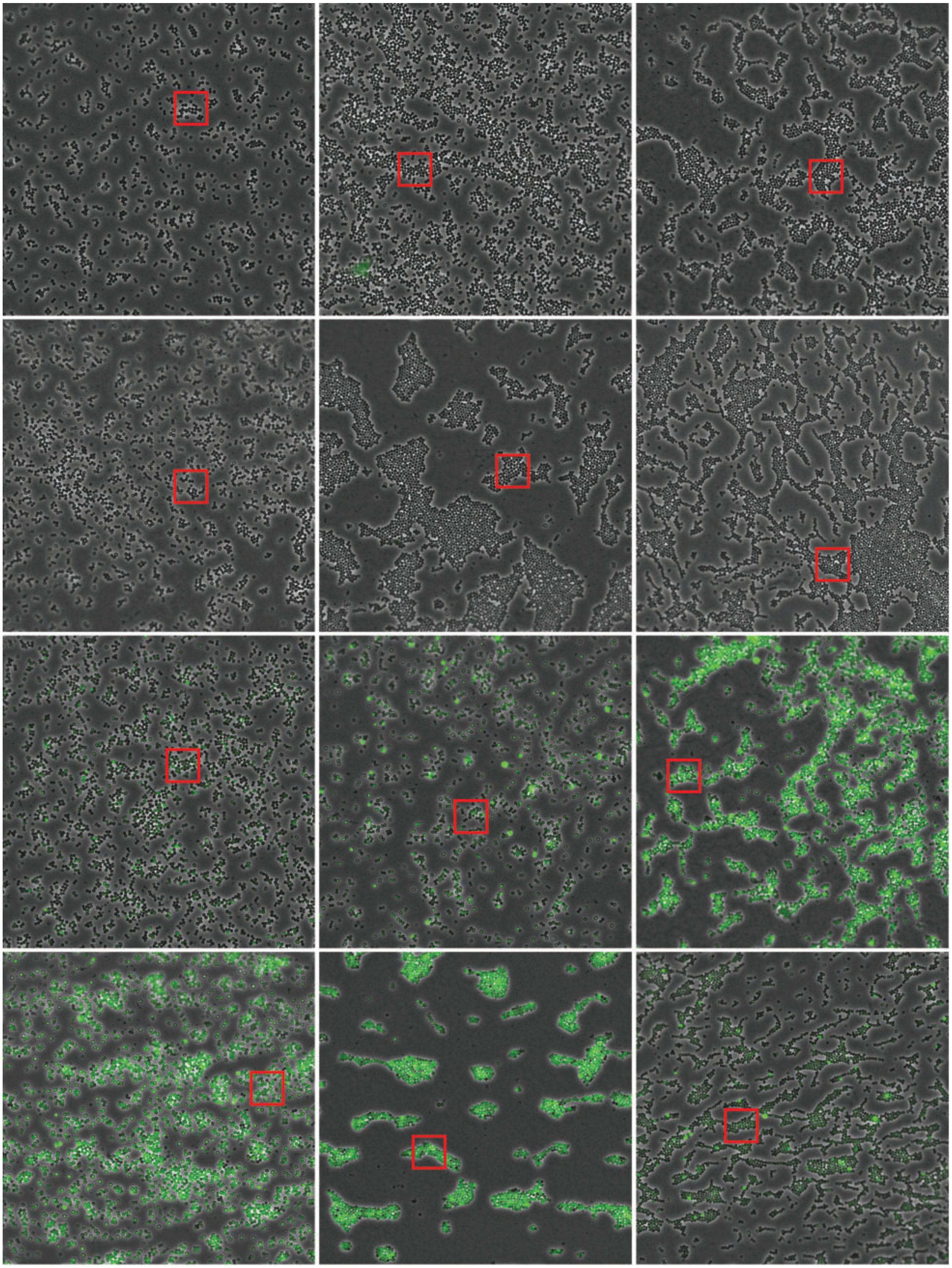

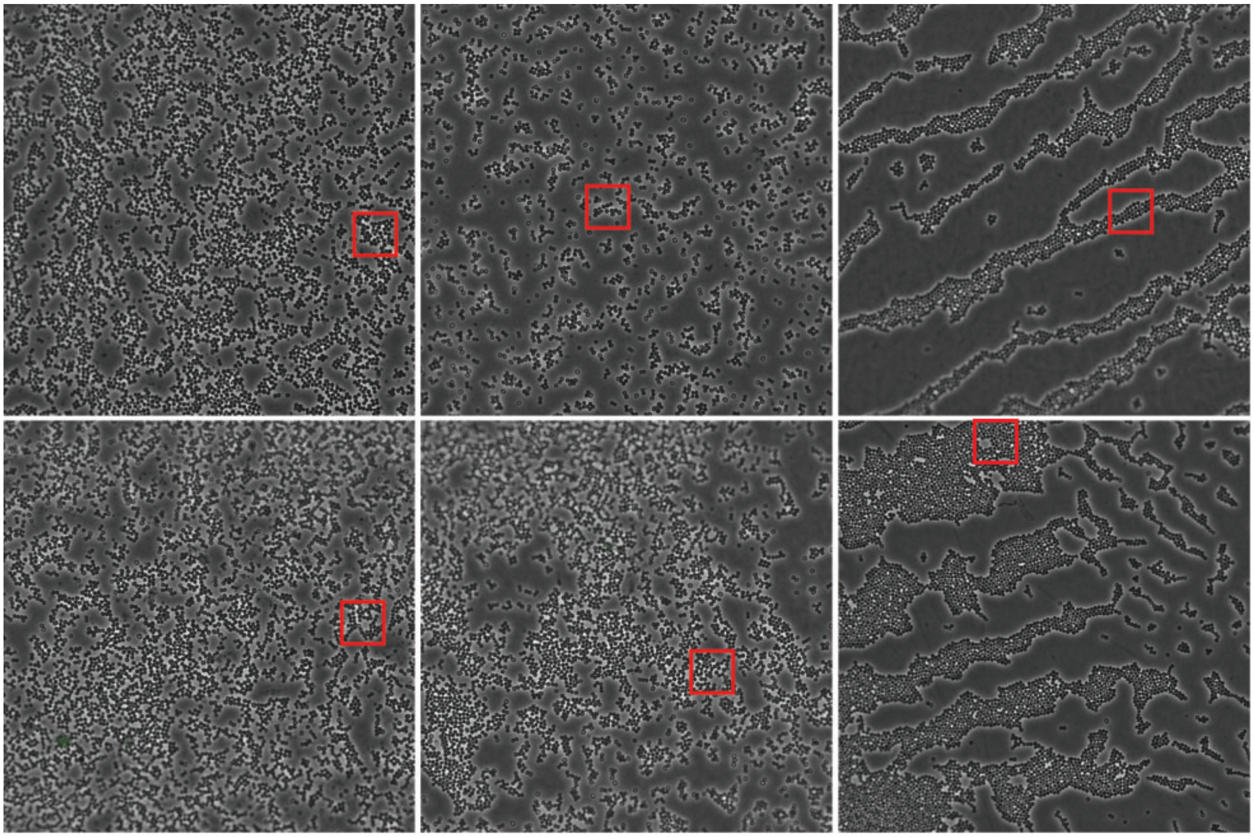

b

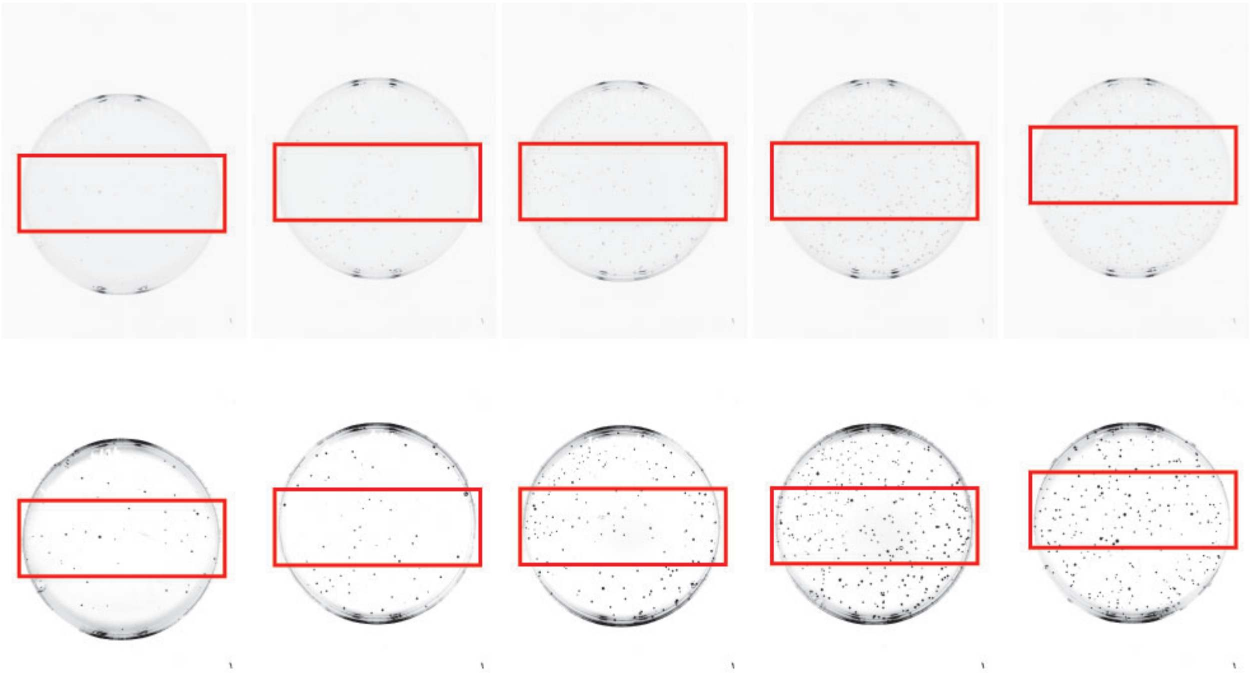

c

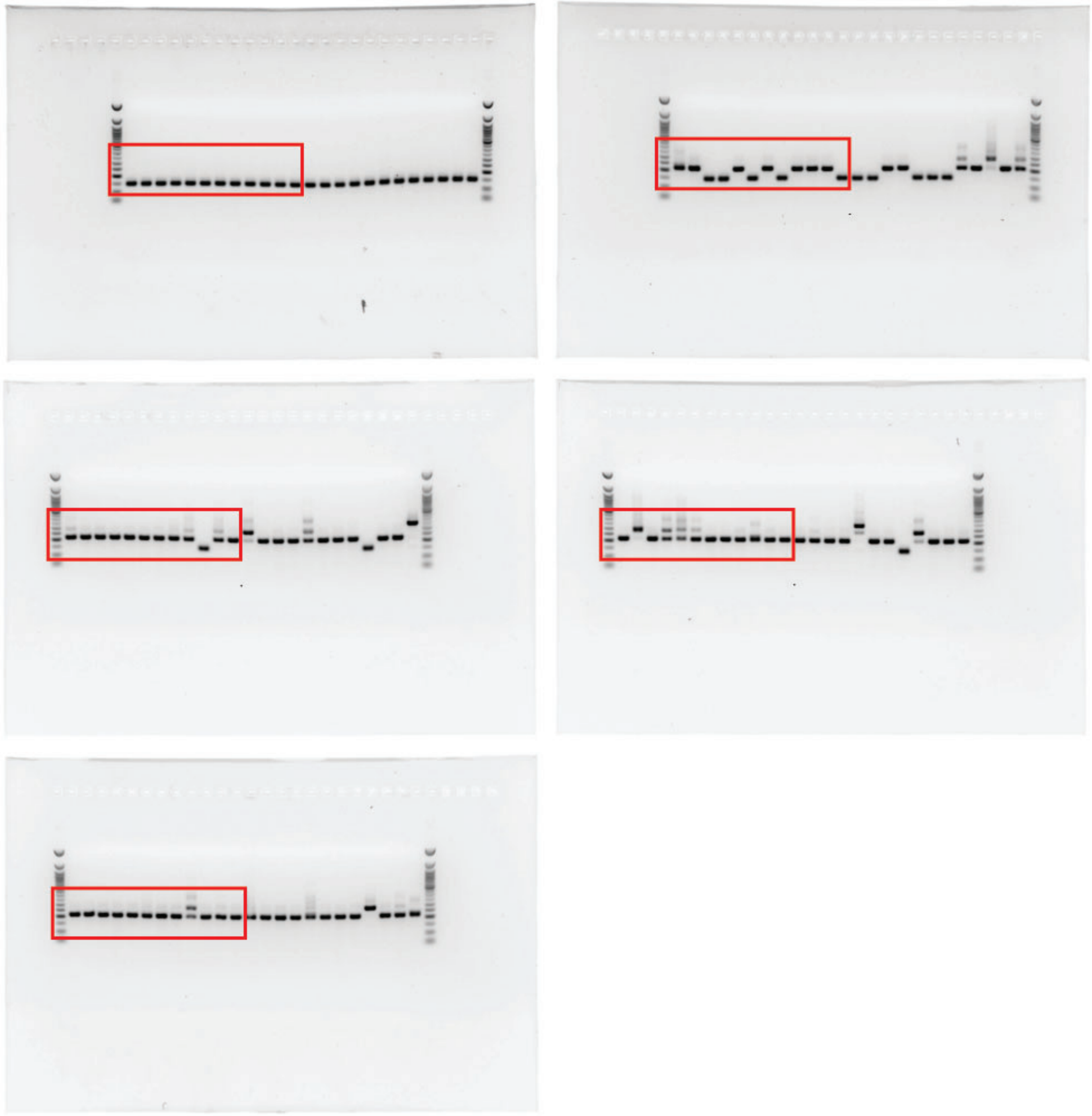

d

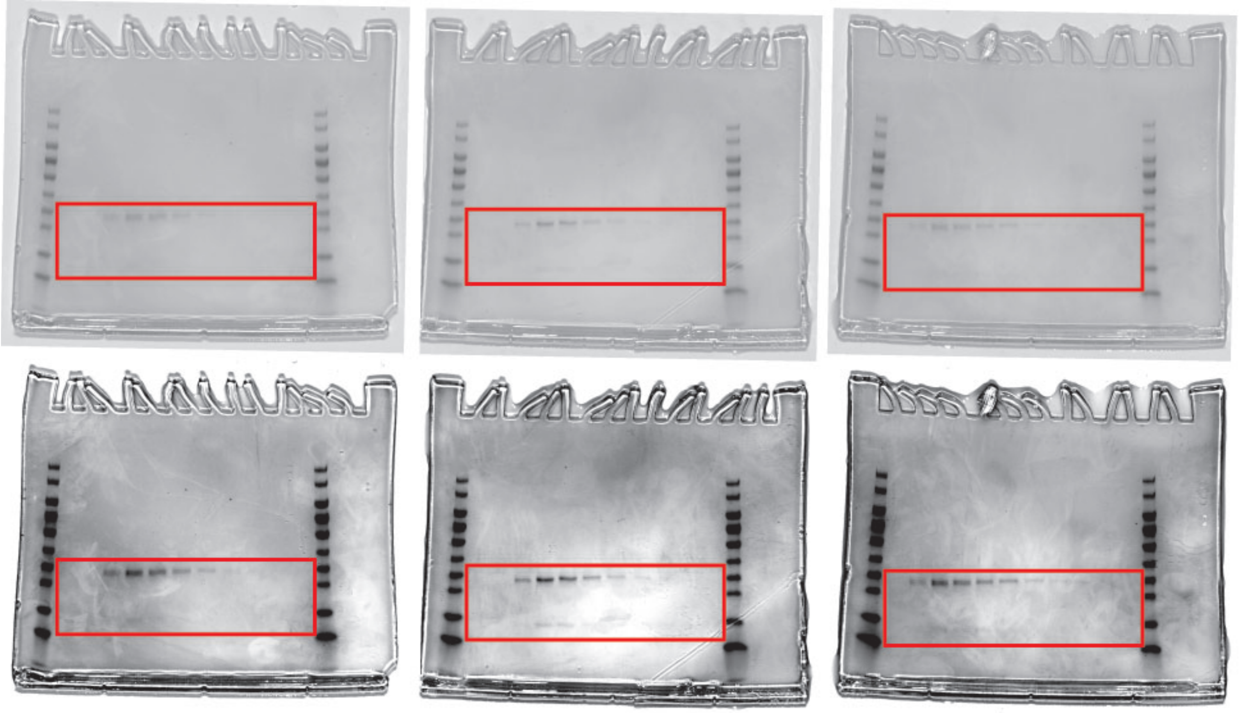

e

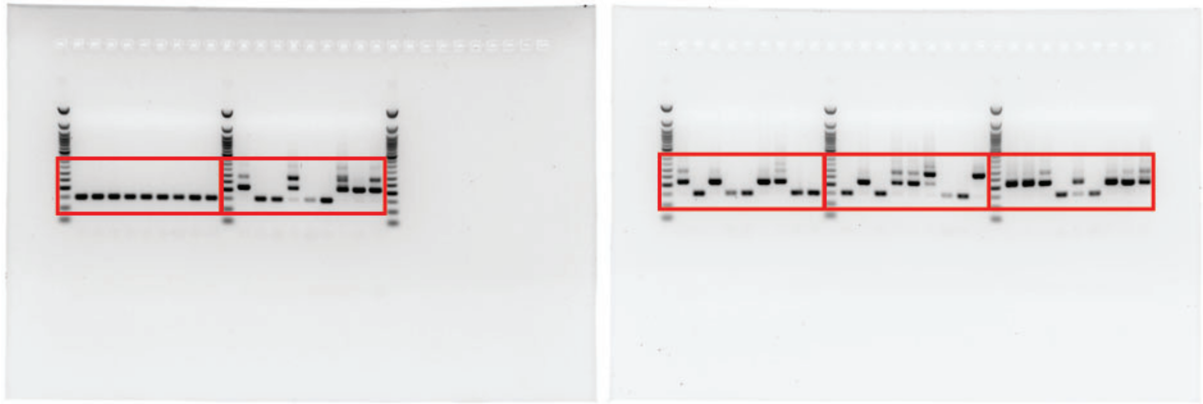

f

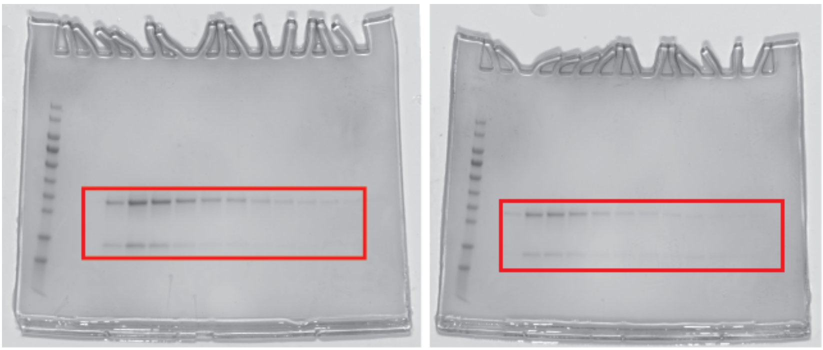

**Supplementary Fig. 2 | Source images presented in this study. a** Uncropped images shown in Fig. 1b of stacked phase contrast (greyscale) and mNG (green, pixel values adjusted to min. 600, max. 3000 using Fiji) channels. **b** Uncropped images shown in Fig. 4a. Top row shows unedited images, and bottom

row shows images with pixel values adjusted to min. 50000 and max. 62000 using Fiji. **c** Uncropped images shown in Fig. 4b. The ladder is a Quick-Load Purple 50 bp DNA Ladder (NEB, N0556). **d** Uncropped images shown in Supplementary Fig. 12c. Top row shows unedited images, and bottom row shows images with pixel values adjusted to min. 15000 and max. 30000 using Fiji. The ladder is a PageRuler Prestained 10 to 180 kDa Protein Ladder (ThermoFisher, 26616). **e** Uncropped images shown in Supplementary Fig. 16b. The ladder is a Quick-Load Purple 50 bp DNA Ladder (NEB, N0556). **f** Uncropped images shown in Supplementary Fig. 18c. The ladder is a PageRuler Prestained 10 to 180 kDa Protein Ladder (ThermoFisher, 26616). Presented parts are highlighted with a red box (**a**–**f**).

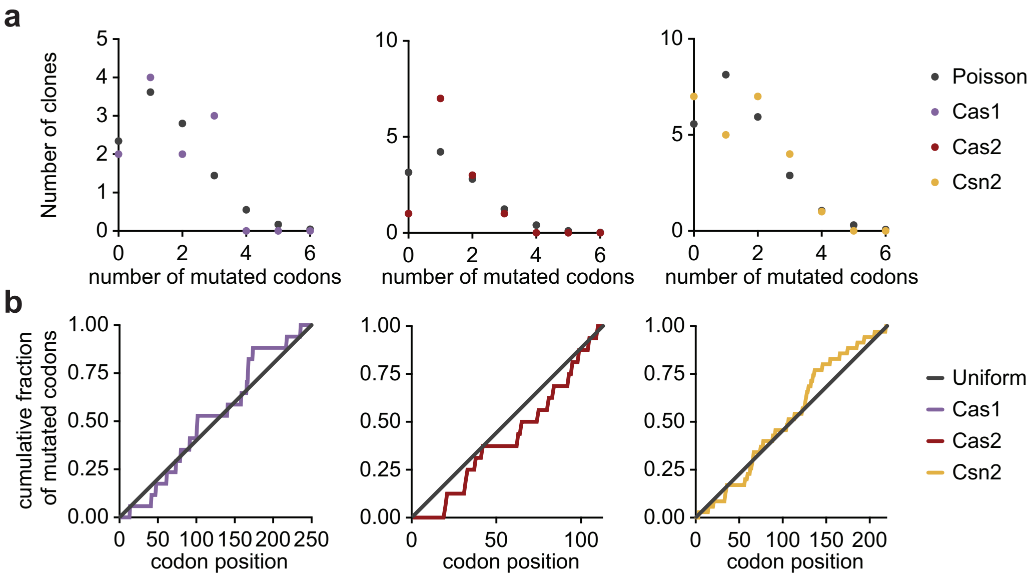


**Supplementary Fig. 3 | Quality control of libraries. a**, **b** Number of mutated codons per clone compared to Poisson distribution(**a**) and distribution of mutated codons along the primary sequence compared to uniform distribution (**b**), based on a sample of the unselected libraries of Cas1 (residues 1–250, *n* = 11), Cas2 (*n* = 12) and Csn2 (*n* = 24).

a

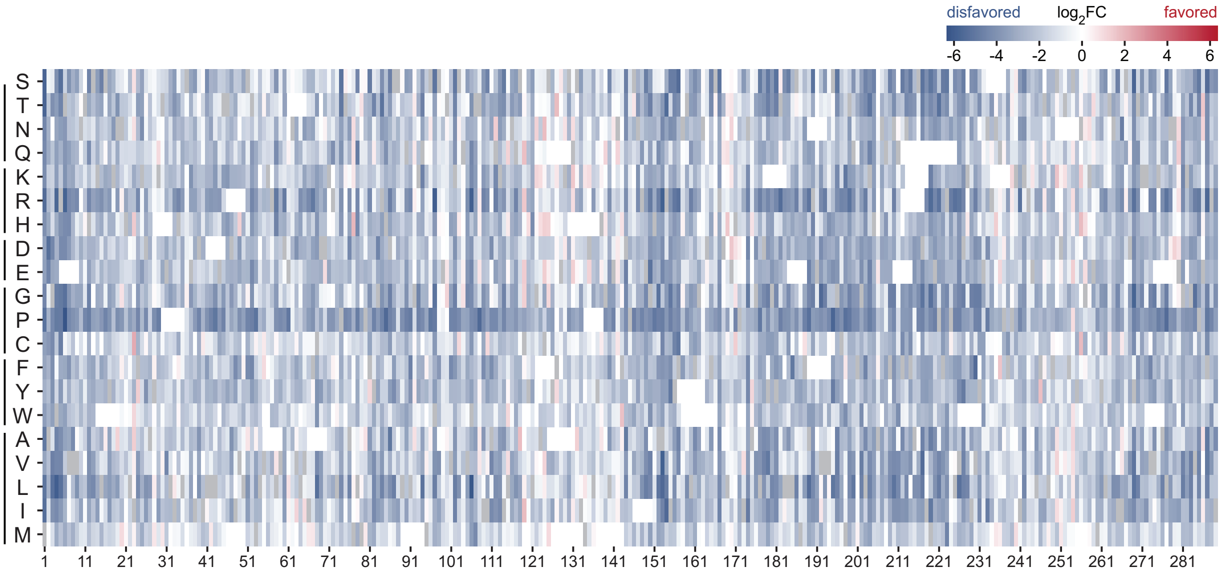
disfavored
log2FC
favored
-6 -4 -2 0 2 4 6
S
T
N
Q
K
R
H
D
E
G
P
C
F
Y
W
A
V
L
I
M
polar
pos.
neg.
special
aromatic
aliphatic
1 11 21 31 41 51 61 71 81 91 101 111 121 131 141 151 161 171 181 191 201 211 221 231 241 251 261 271 281

b

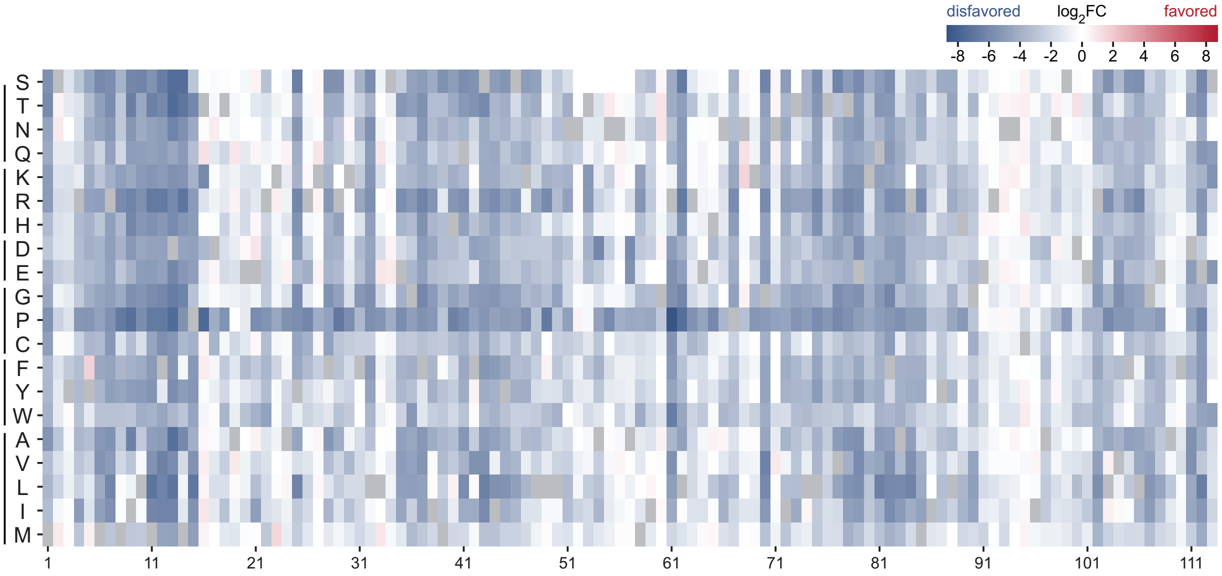
disfavored
log2FC
favored
-8 -6 -4 -2 0 2 4 6 8
S
T
N
Q
K
R
H
D
E
G
P
C
F
Y
W
A
V
L
I
M
polar
pos.
neg.
special
aromatic
aliphatic
1 11 21 31 41 51 61 71 81 91 101 111

c

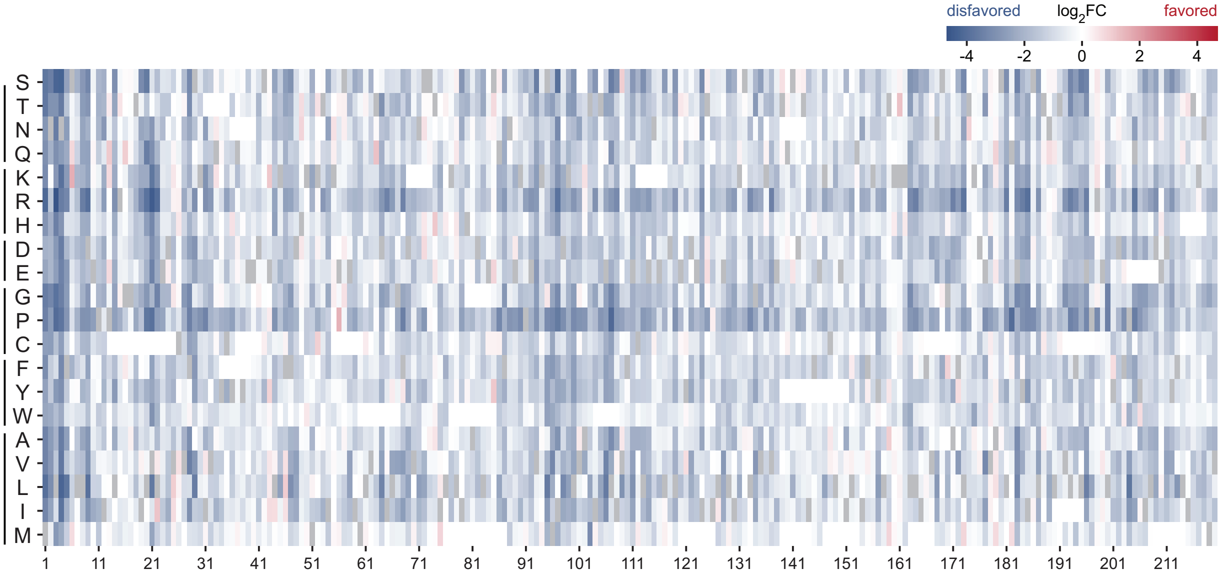
disfavored
log2FC
favored
-4 -2 0 2 4
S
T
N
Q
K
R
H
D
E
G
P
C
F
Y
W
A
V
L
I
M
polar
pos.
neg.
special
aromatic
aliphatic
1 11 21 31 41 51 61 71 81 91 101 111 121 131 141 151 161 171 181 191 201 211

**Supplementary Fig. 4 | Heatmaps of global differential selection across Cas1, Cas2 and Csn2.** Global differential selection of amino acids across Cas1 (**a**), Cas2 (**b**) and Csn2 (**c**) according to DMS. Columns along the x-axis represent the positions in the protein sequence, and rows along the y-axis show the differential selection of each amino acid at that positions, colored by $\log_2$ fold change ($\log_2$FC).

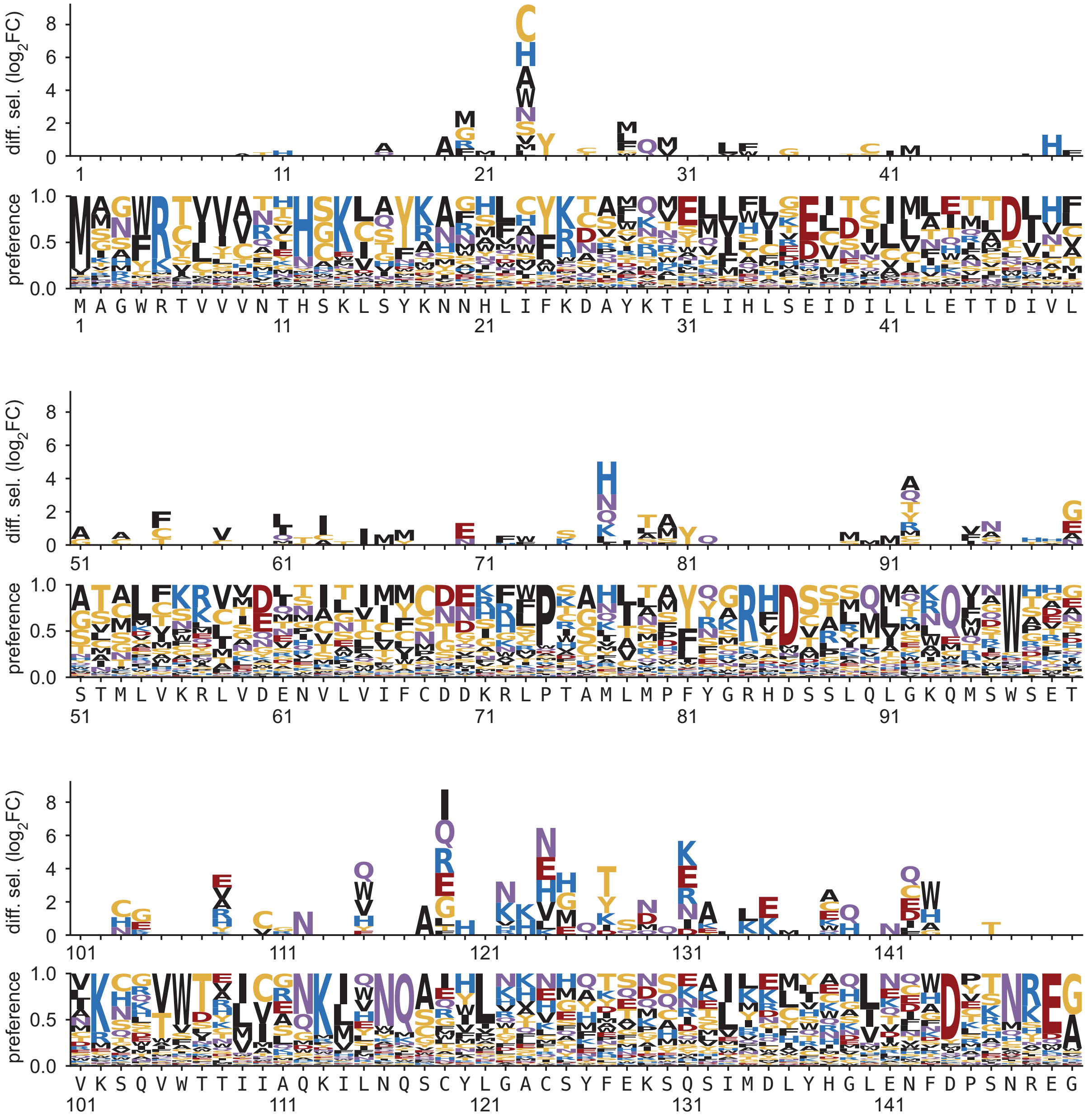


**Supplementary Fig. 5 | Positive differential selection and amino acid preferences of Cas1.** Positive differential selection (top) and amino acid preferences (bottom) of Cas1 as determined by DMS. Figure continues to next page.

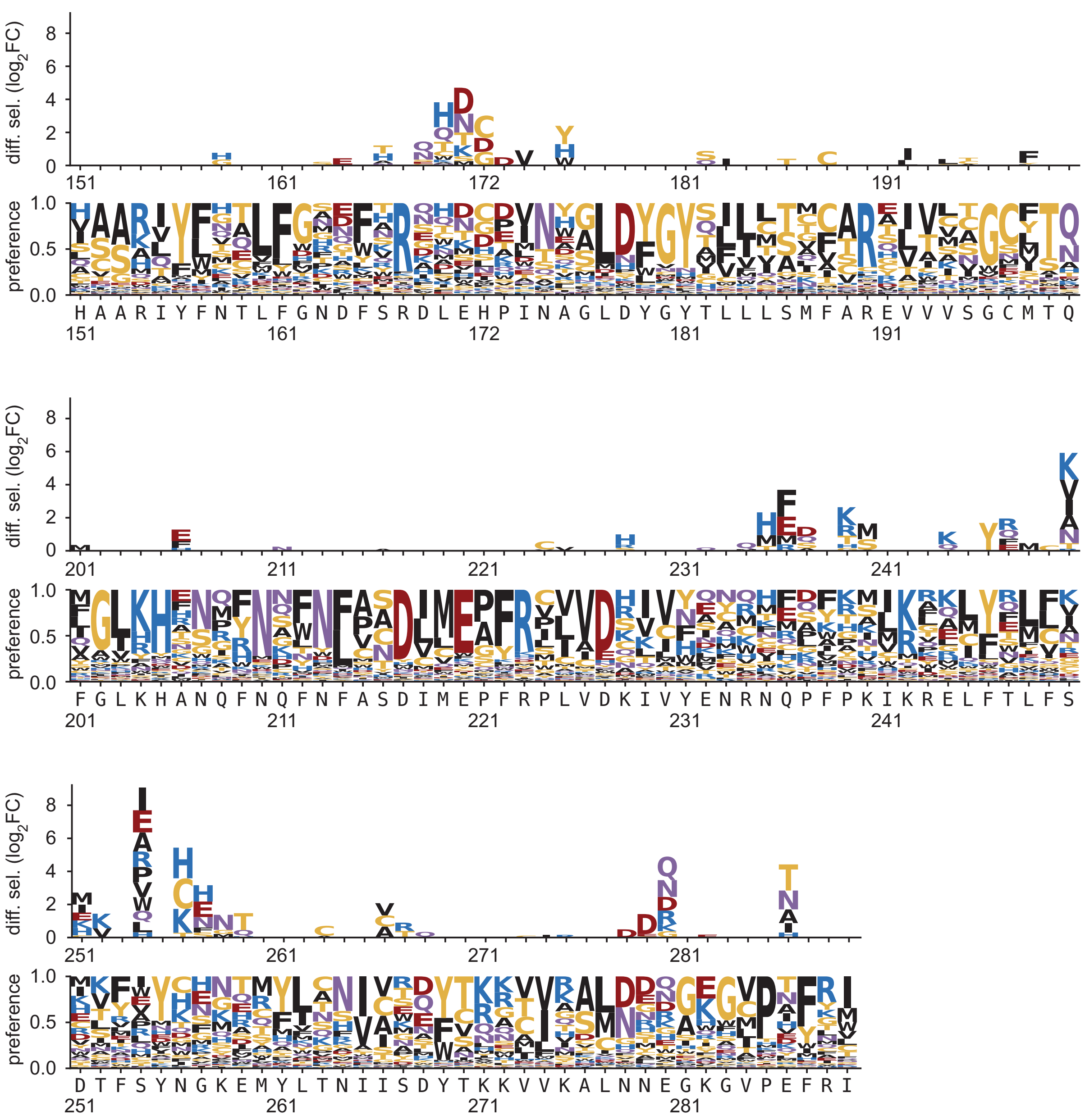


**Supplementary Fig. 5 | Continued.**

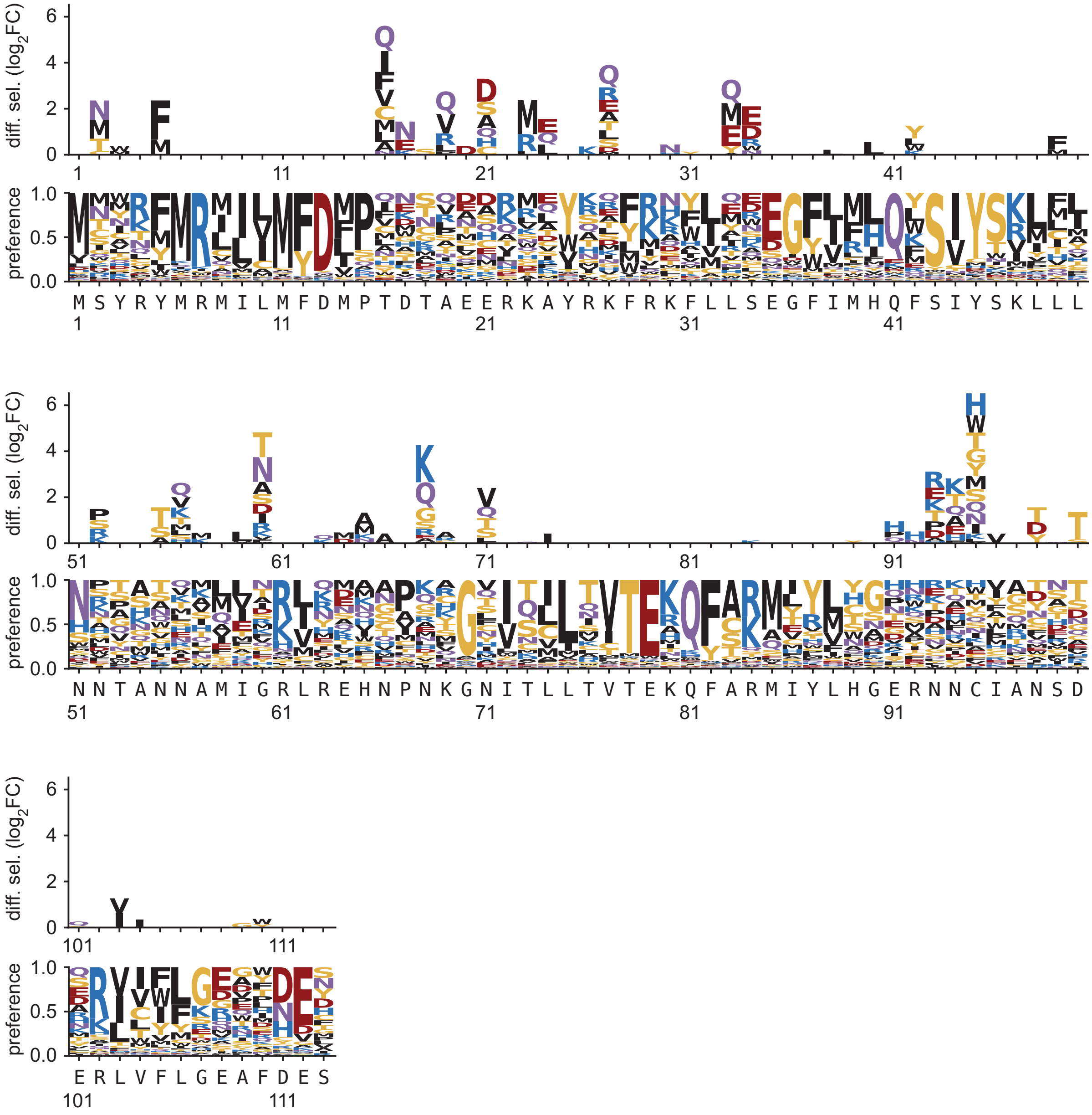


**Supplementary Fig. 6 | Positive differential selection and amino acid preferences of Cas2.** Positive differential selection (top) and amino acid preferences (bottom) of Cas2 as determined by DMS.

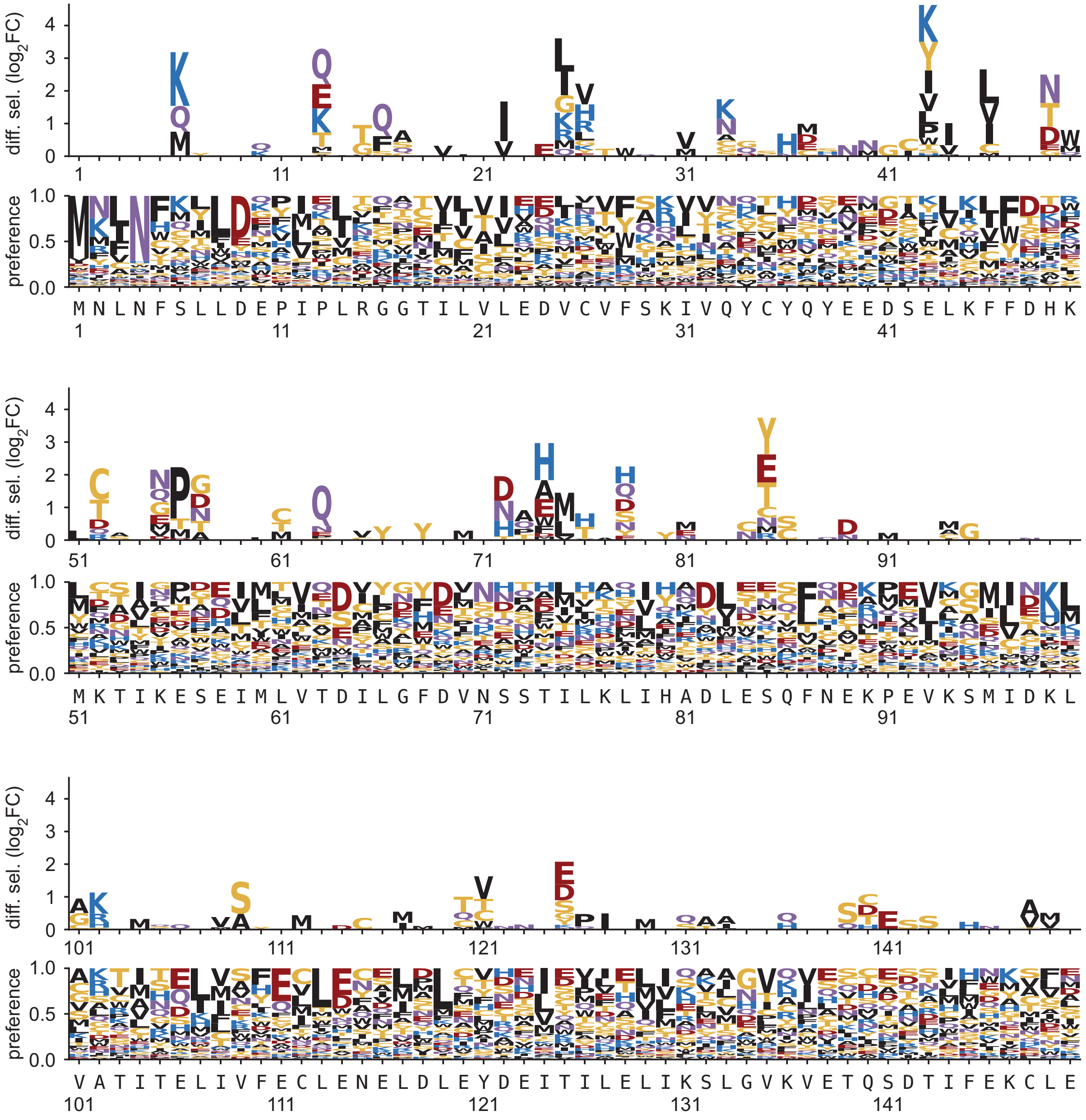


**Supplementary Fig. 7 | Positive differential selection and amino acid preferences of Csn2.** Positive differential selection (top) and amino acid preferences (bottom) of Csn2 as determined by DMS. Figure continues to next page.

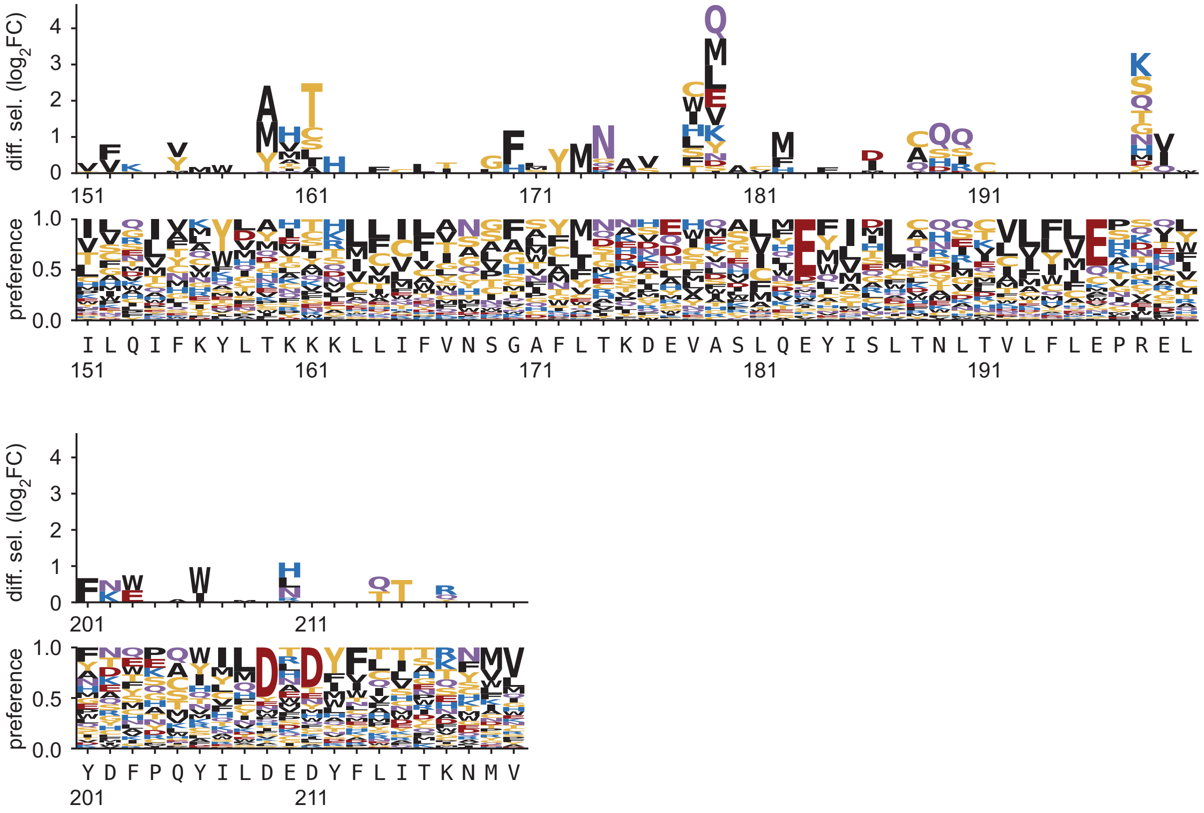


**Supplementary Fig. 7 | Continued.**

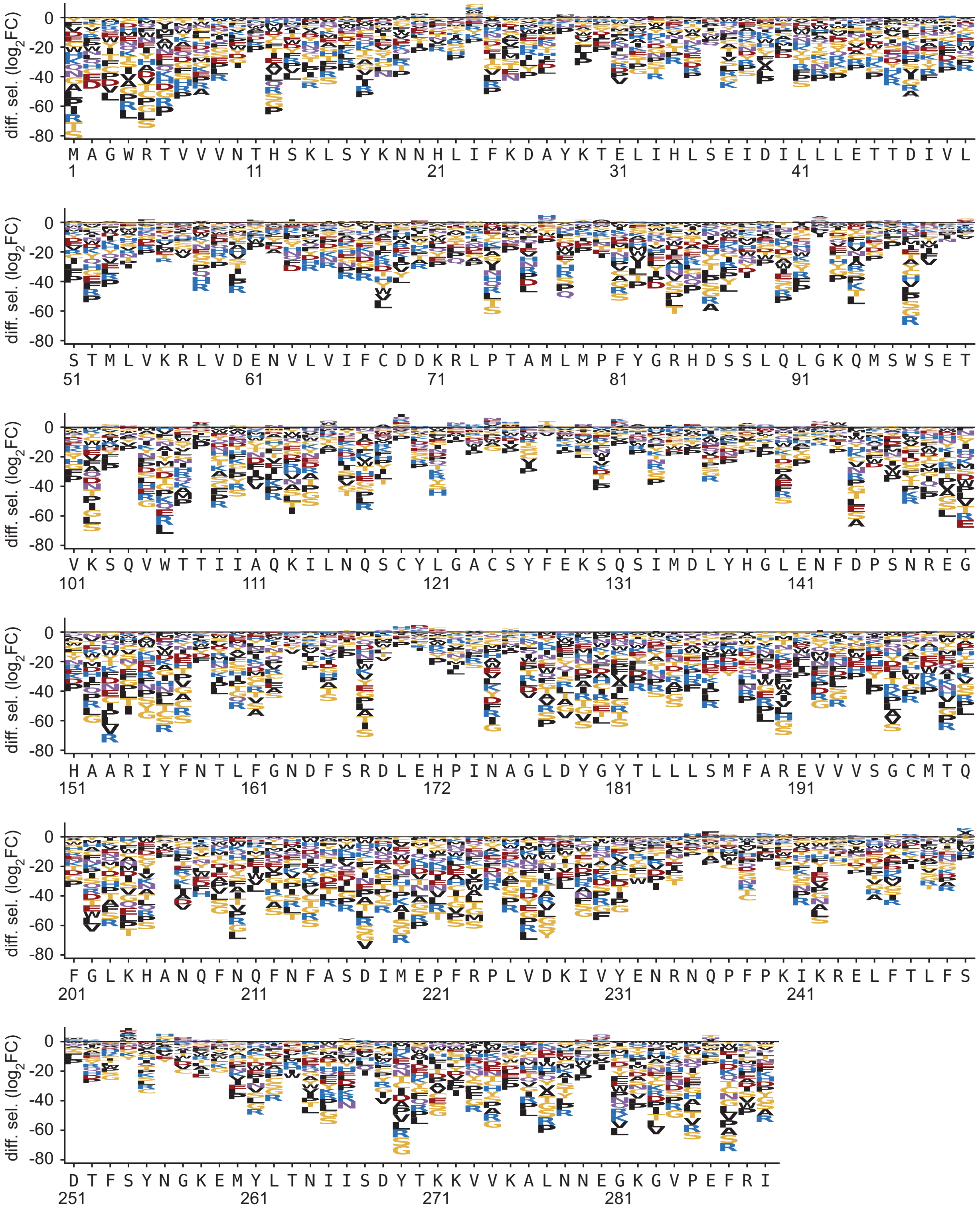


**Supplementary Fig. 8 | Positive and negative differential selection of Cas1.**

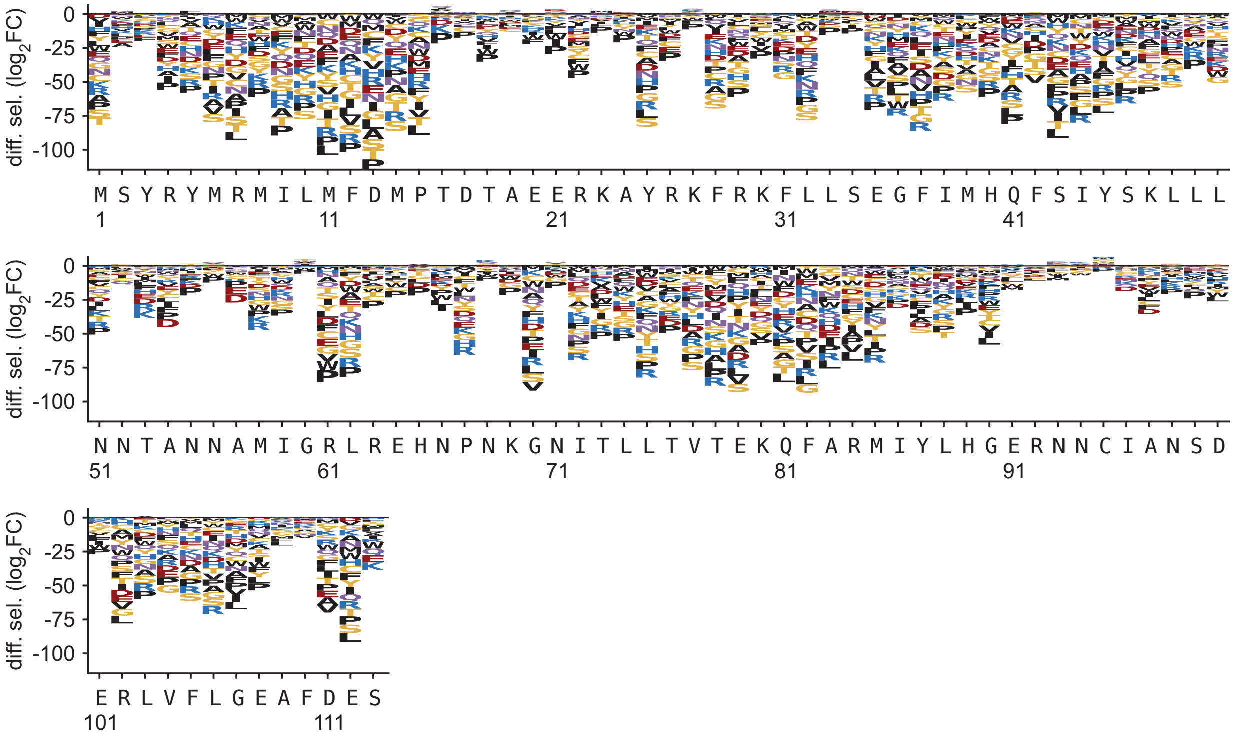


**Supplementary Fig. 9 | Positive and negative differential selection of Cas2.**

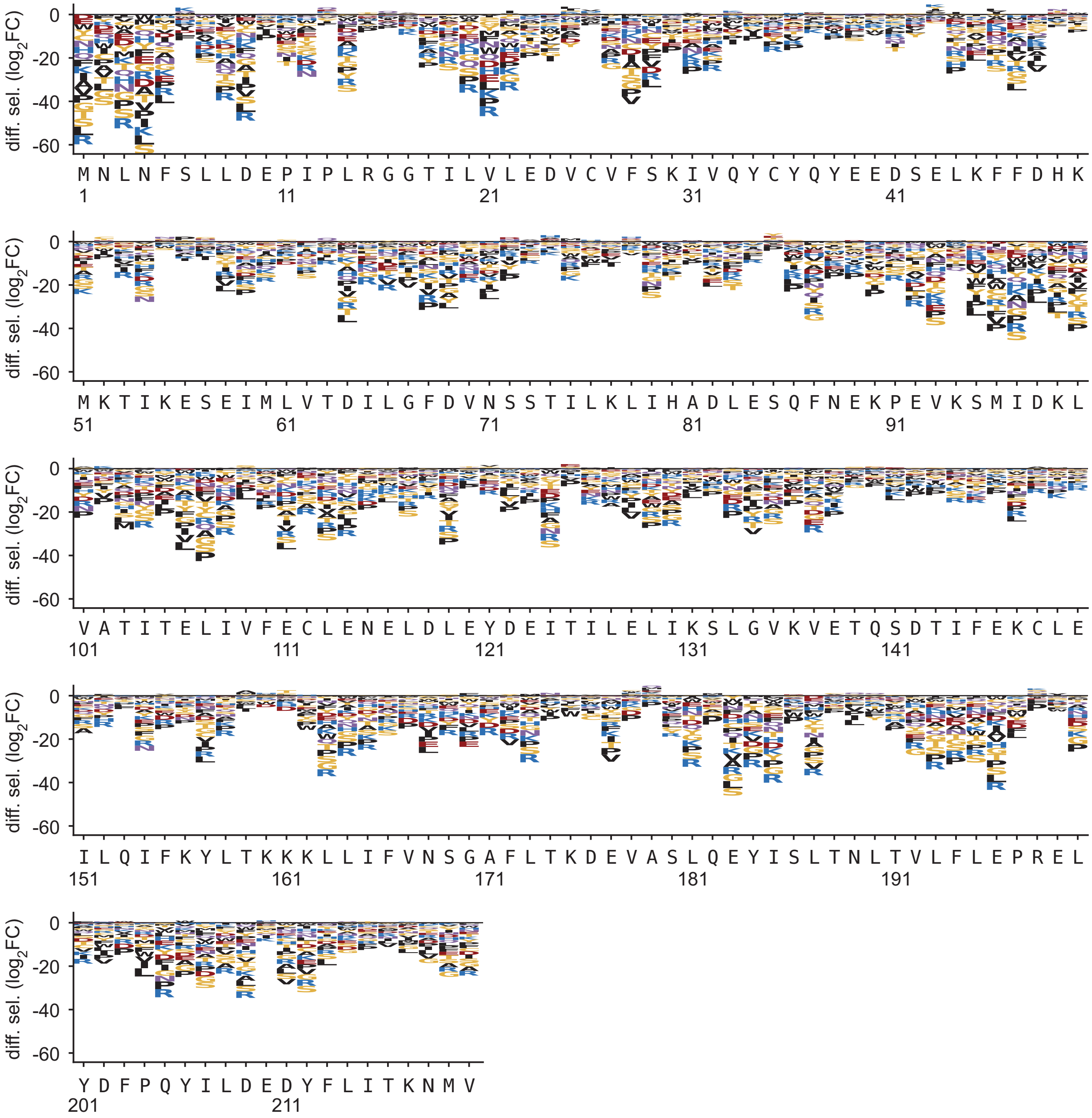


**Supplementary Fig. 10 | Positive and negative differential selection of Csn2.**

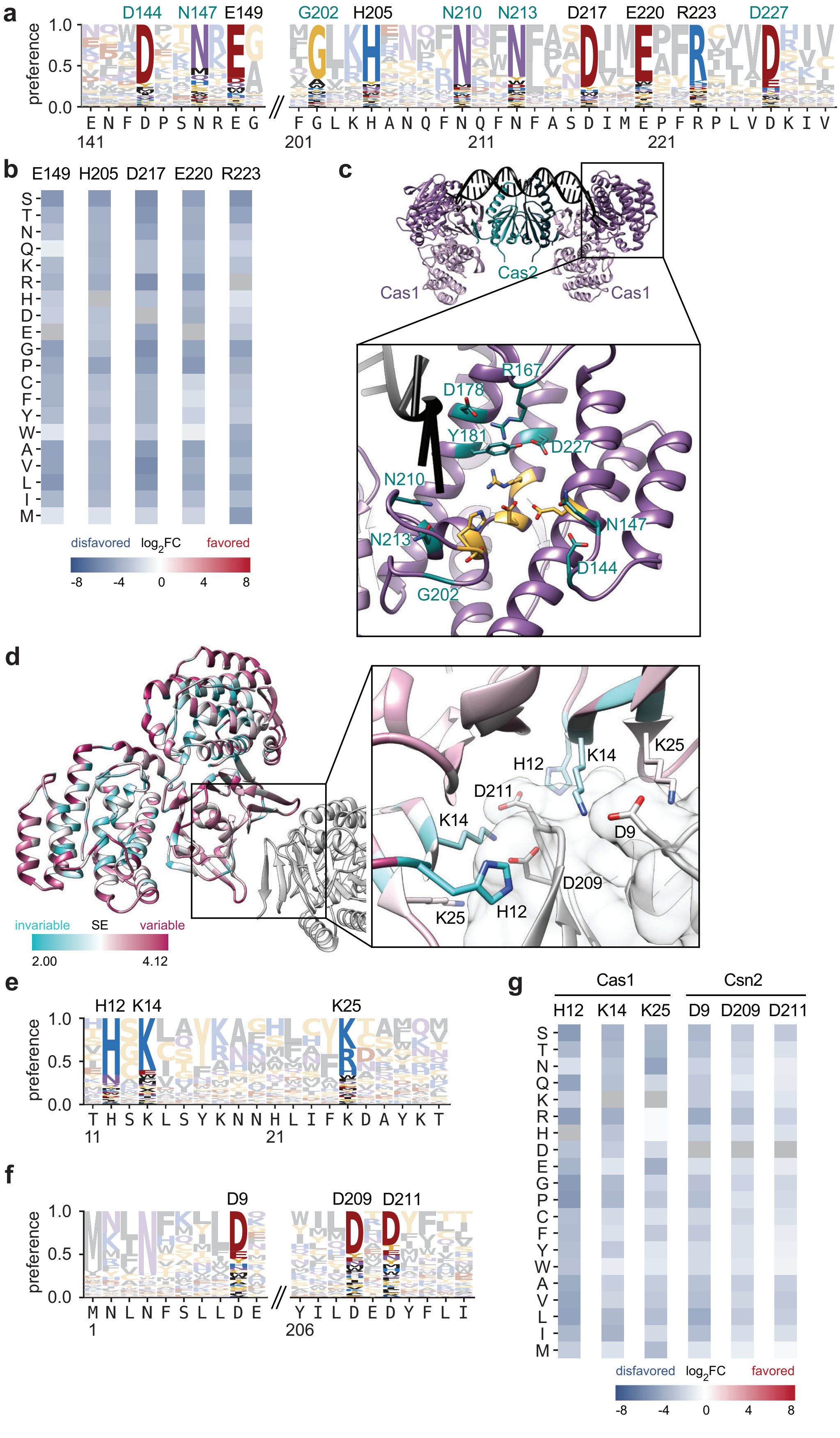
a
D144 N147 E149
G202 H205 N210 N213 D217 E220 R223 D227
preference
1.0
0.5
0.0
E N F D P S N R E G
F G L K H A N Q F N Q F N F A S D I M E P F R P L V D K I V
141
201
211
221
b
E149 H205 D217 E220 R223
S T N Q K R H D E G P C F Y W A V L I M
disfavored log2FC favored
-8 -4 0 4 8
c
Cas2
Cas1
Cas1
R167
D178
Y181
D227
N210
N147
N213
D144
G202
d
invariable SE variable
2.00 4.12
H12
K14
K25
D211
K14
D9
D209
K25
H12
e
H12 K14 K25
preference
1.0
0.5
0.0
T H S K L S Y K N N H L I F K D A Y K T
11
21
f
D9 D209 D211
preference
1.0
0.5
0.0
M N L N F S L L D E
Y I L D E D Y F L I
1
206
g
Cas1 Csn2
H12 K14 K25 D9 D209 D211
S T N Q K R H D E G P C F Y W A V L I M
disfavored log2FC favored
-8 -4 0 4 8

**Supplementary Fig. 11 | Validation of DMS results. a** Amino acid preference of indicated Cas1 regions. Known catalytic residues are labeled in black and other highly preferred residues in the catalytic pocket in green. **b** Known Cas1 active site residues (columns) and differential selection of all possible amino acids at these positions (rows), colored by $log_2$ fold change ($log_2FC$). **c** AlphaFold3 model of the Cas1-Cas2 integrase complex. The inset shows the known catalytic residues in orange and other highly preferred residues in green. **d** AlphaFold3 model of a Cas1 dimer, colored by Shannon Entropy (SE), in complex with Csn2 (grey). The inset shows highly preferred residues at the protein-protein interface. **e**, **f** Amino acid preference of indicated Cas1 (**e**) and Csn2 (**f**) regions. Interacting residues are highlighted. **g** Differential selection of Cas1 and Csn2 residues that engage in protein-protein interactions, displayed as in **b**.

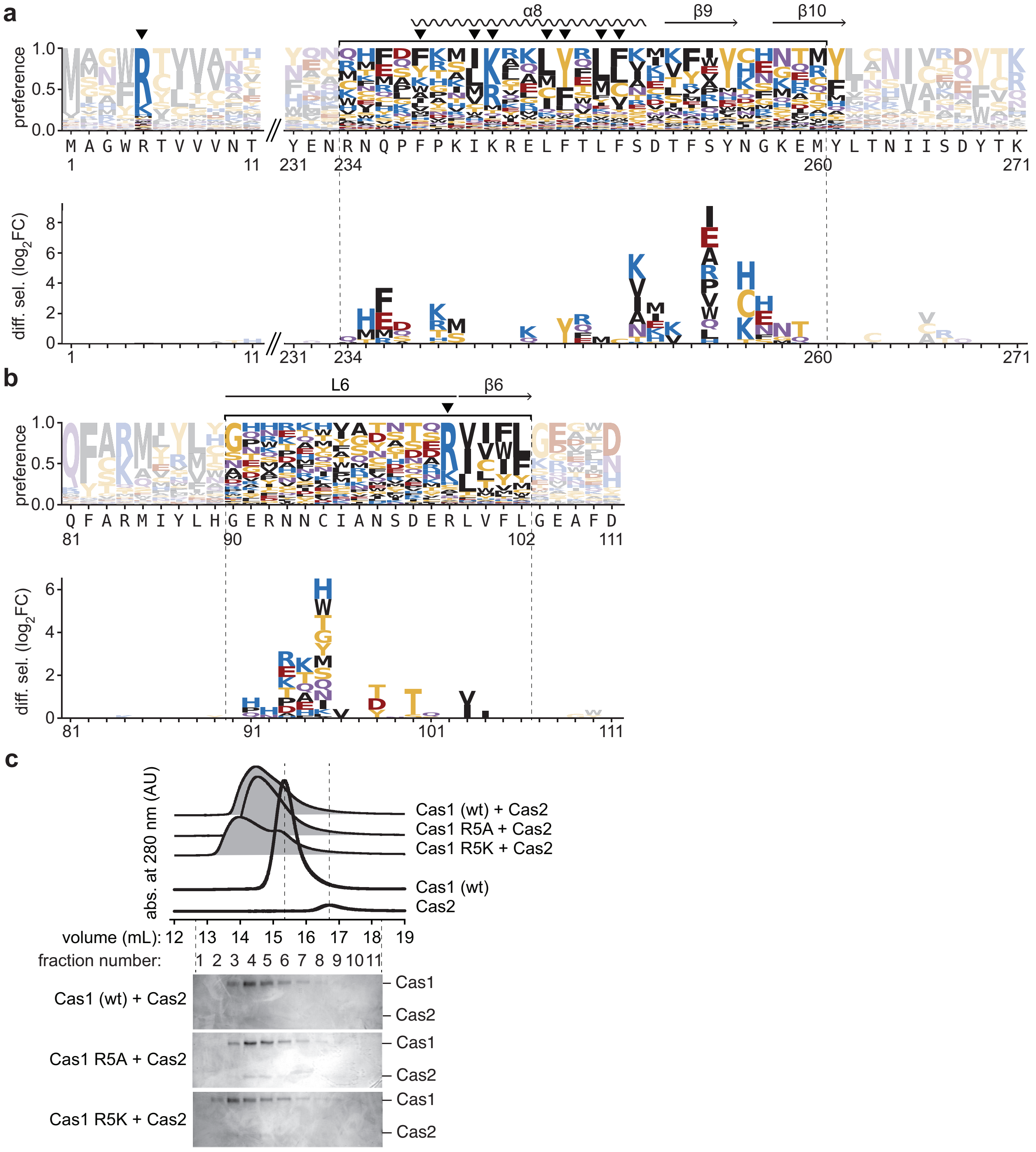


**Supplementary Fig. 12 | Cas1-Cas2 interaction. a**, **b** Amino acid preference (top) and positive differential selection (bottom) of indicated Cas1 (**a**) and Cas2 (**b**) regions. Select secondary structure elements are indicated, and relatively conserved residues are marked by a black arrowhead. **c** Interaction of indicated Cas1 variants (2 equiv.) and Cas2 as determined by analytical SEC (top), and Coomassie blue SDS-PAGE analysis of corresponding elution fractions (bottom). Elution profiles of individual protein standards are shown.

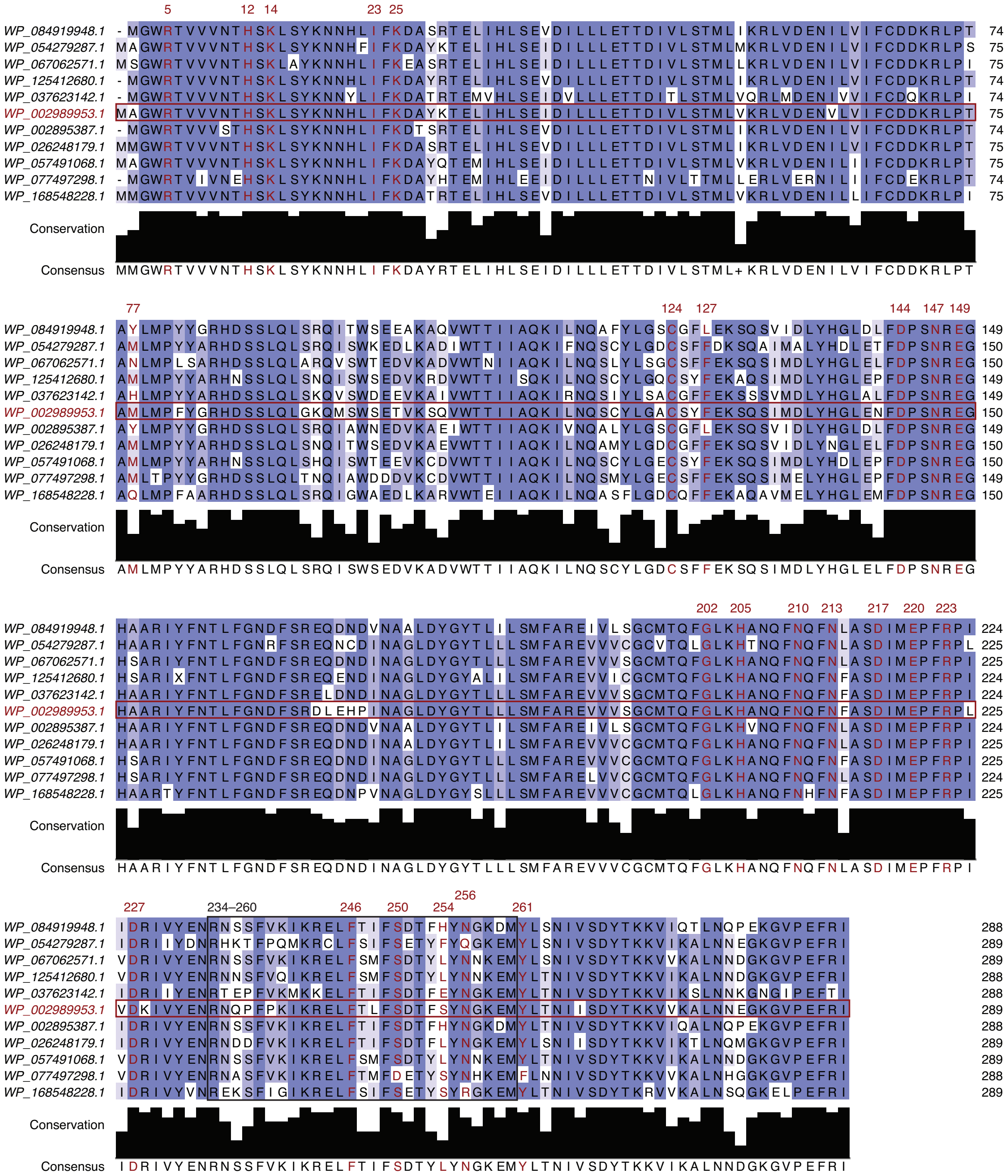


**Supplementary Fig. 13 | Alignment of Cas1 protein sequences.** Alignment of Cas1 protein sequences, colored by sequence identity. The sequence of *S. pyogenes* Cas1 is highlighted with a red box, select positions are highlighted in red font, and residues 234–260 are highlighted with a black box.

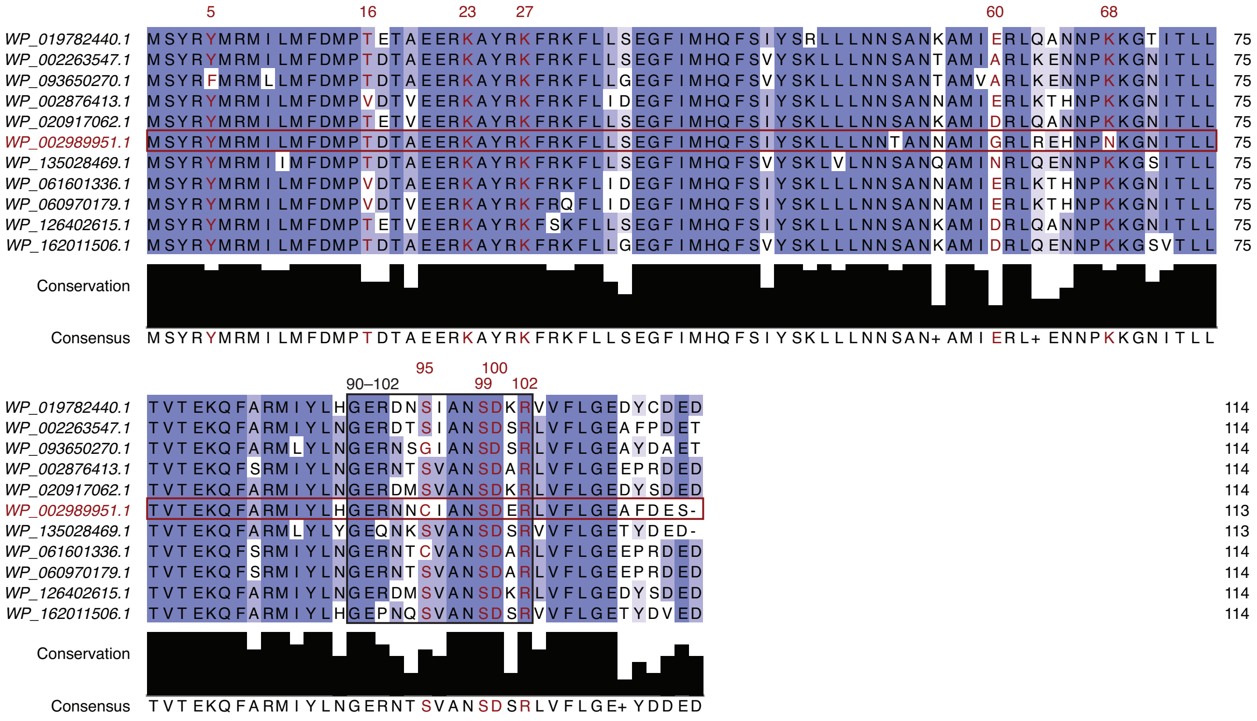

**Supplementary Fig. 14 | Alignment of Cas2 protein sequences.** Alignment of Cas2 protein sequences, colored by sequence identity. The sequence of *S. pyogenes* Cas2 is highlighted with a red box, select positions are highlighted in red font, and residues 90–102 are highlighted with a black box.

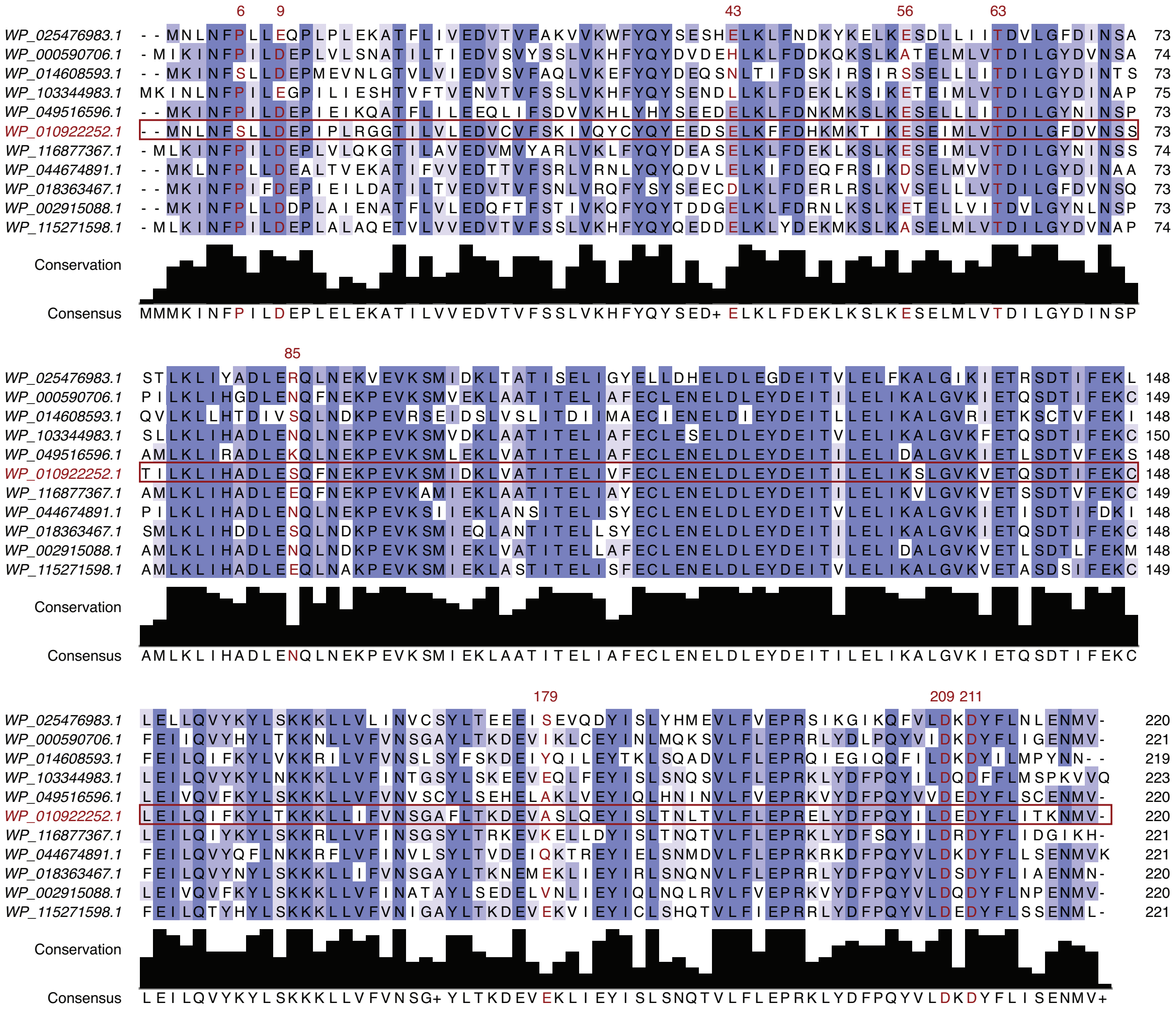


**Supplementary Fig. 15 | Alignment of Csn2 protein sequences.** Alignment of Csn2 protein sequences, colored by sequence identity. The sequence of *S. pyogenes* Csn2 is highlighted with a red box and select positions are highlighted in red font.

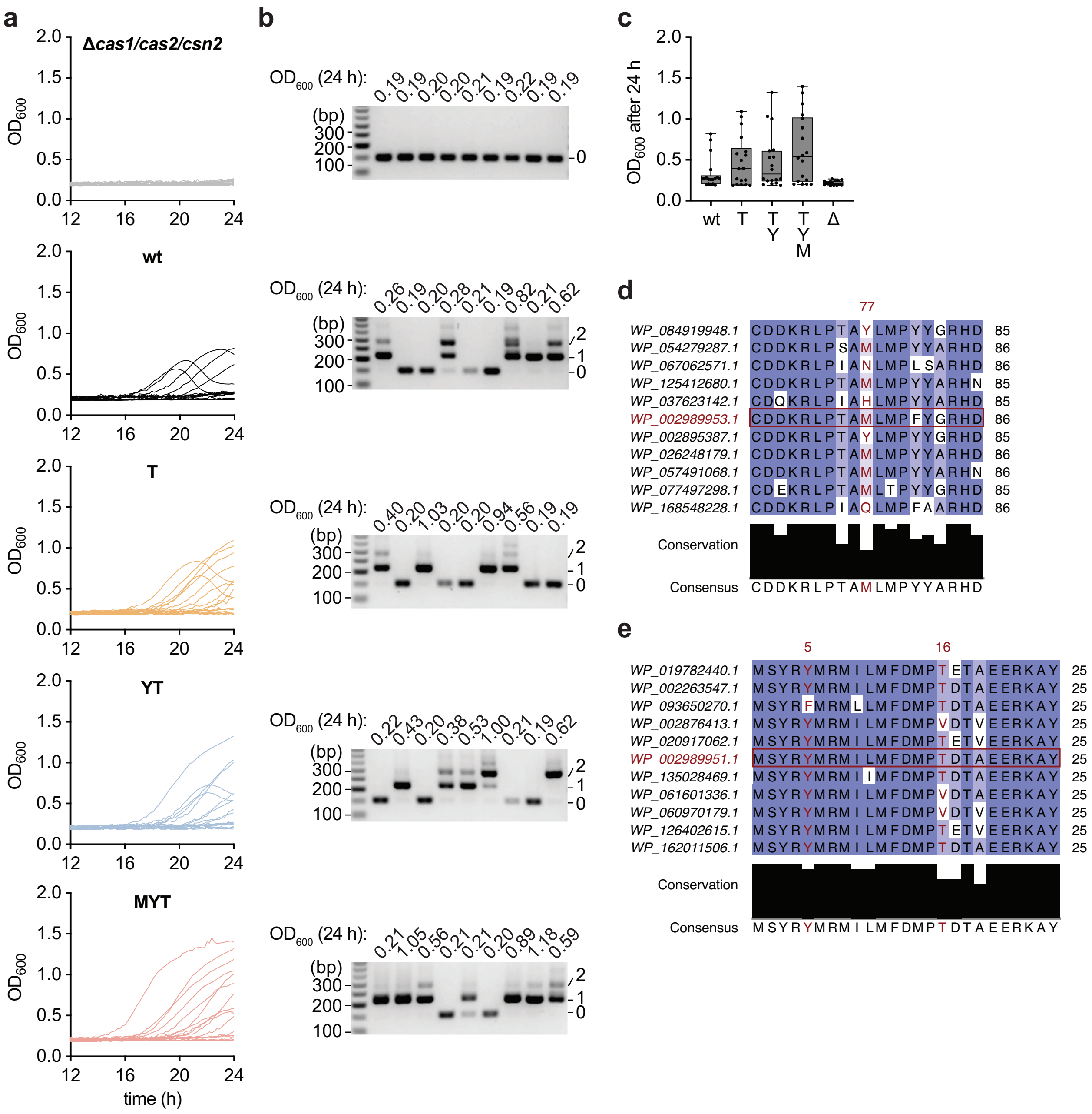


**Supplementary Fig. 16 | Phage immunity and conservation. a** Growth curves after lytic infection of *S. aureus* strains, carrying indicated variants of the type II-A CRISPR-Cas system with a naive CRISPR array (single repeat) on a plasmid, with phage ΦNM4γ4, measured as $OD_{600}$ over time. Shown are data from *n* = 18 individual infections (from six biological replicates, each split into three wells and infected separately) used in Fig. 4d. **b** Agarose gel electrophoresis of PCR amplification of the CRISPR array present in plasmids extracted from nine cultures in **a** after 24 h. The number of spacers in the PCR product (0, 1 or 2) is indicated on the right, and the final $OD_{600}$ is shown at the top of each lane. **c** $OD_{600}$ of the growth curves in **a** after 24 h. Individual data points are shown with box limits representing the interquartile range, whiskers indicating the minimum and maximum, and center lines showing the median. **d**, **e** Alignment of Cas1 (**d**) and Cas2 (**e**) protein sequences, colored by sequence identity. The sequences of *S. pyogenes* are highlighted with red boxes, and the positions with residue changes that support enhanced spacer acquisition are highlighted in red font.

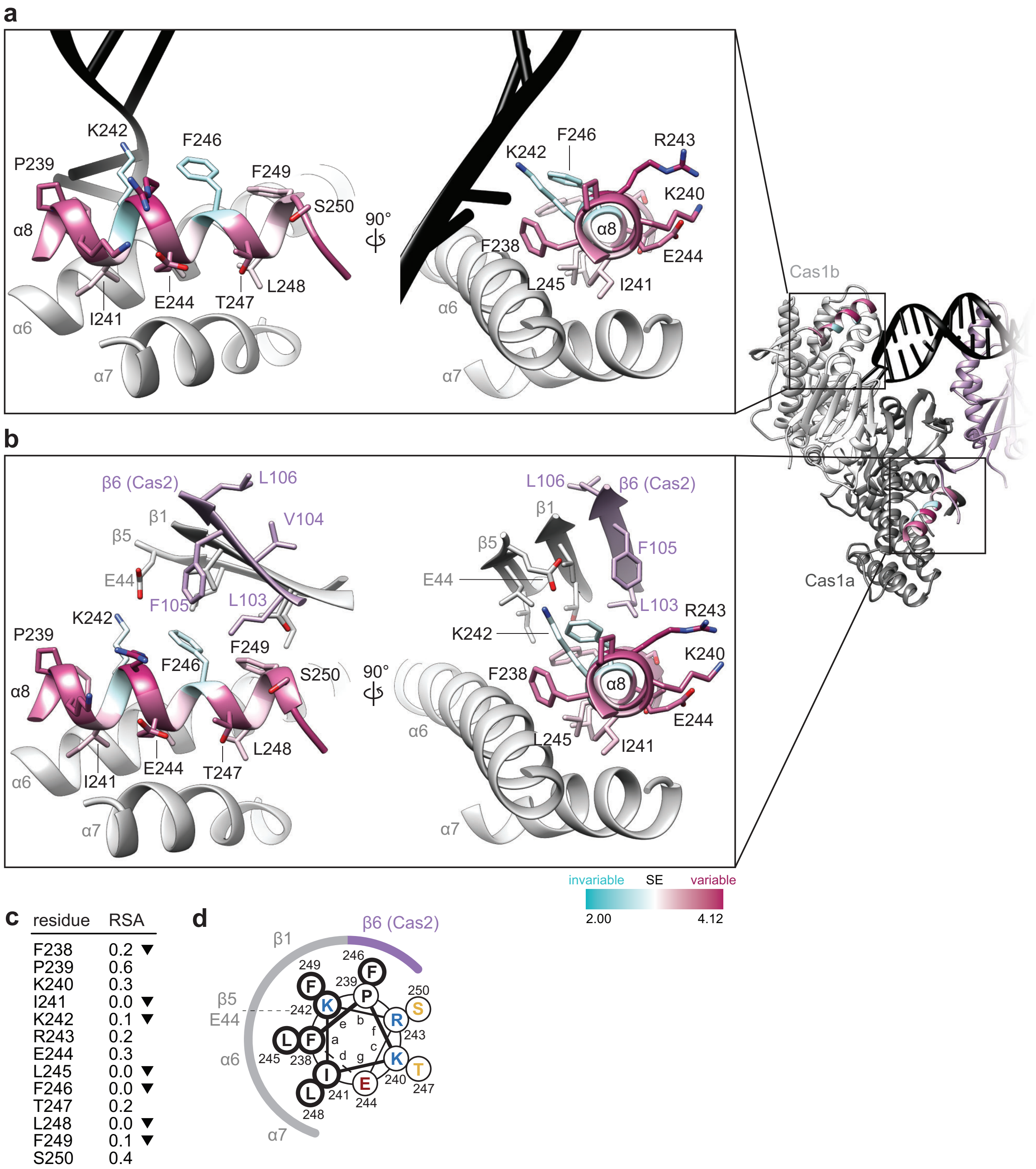


**Supplementary Fig. 17 | Interactions of α8-helices in Cas1 protomers. a**, **b** AlphaFold3 model of Cas1-Cas2 integrase complex. A 22-bp prespacer with 4-nt overhangs is shown in black, the Cas1 dimer is shown in dark (Cas1a) and light (Cas1b) grey, and Cas2 is colored in purple. The insets show orthogonal views of the interactions between the Cas1b α8-helix and the prespacer DNA (**a**) and of the interactions between the Cas1a α8-helix and the Cas2 β6-sheet (purple) (**b**). Cas1 α8-helices are colored by Shannon Entropy, SE. **c** Relative solvent accessibility (RSA) of residues in the Cas1a α8-helix. Select residues are marked with a black arrowhead as in Supplementary Fig. 12. **d** Helical wheel diagram of the Cas1a α8-helix, and schematic representation of interactions with Cas2 (purple) and other parts of Cas1a (grey). Relatively conserved residues are circled in bold.

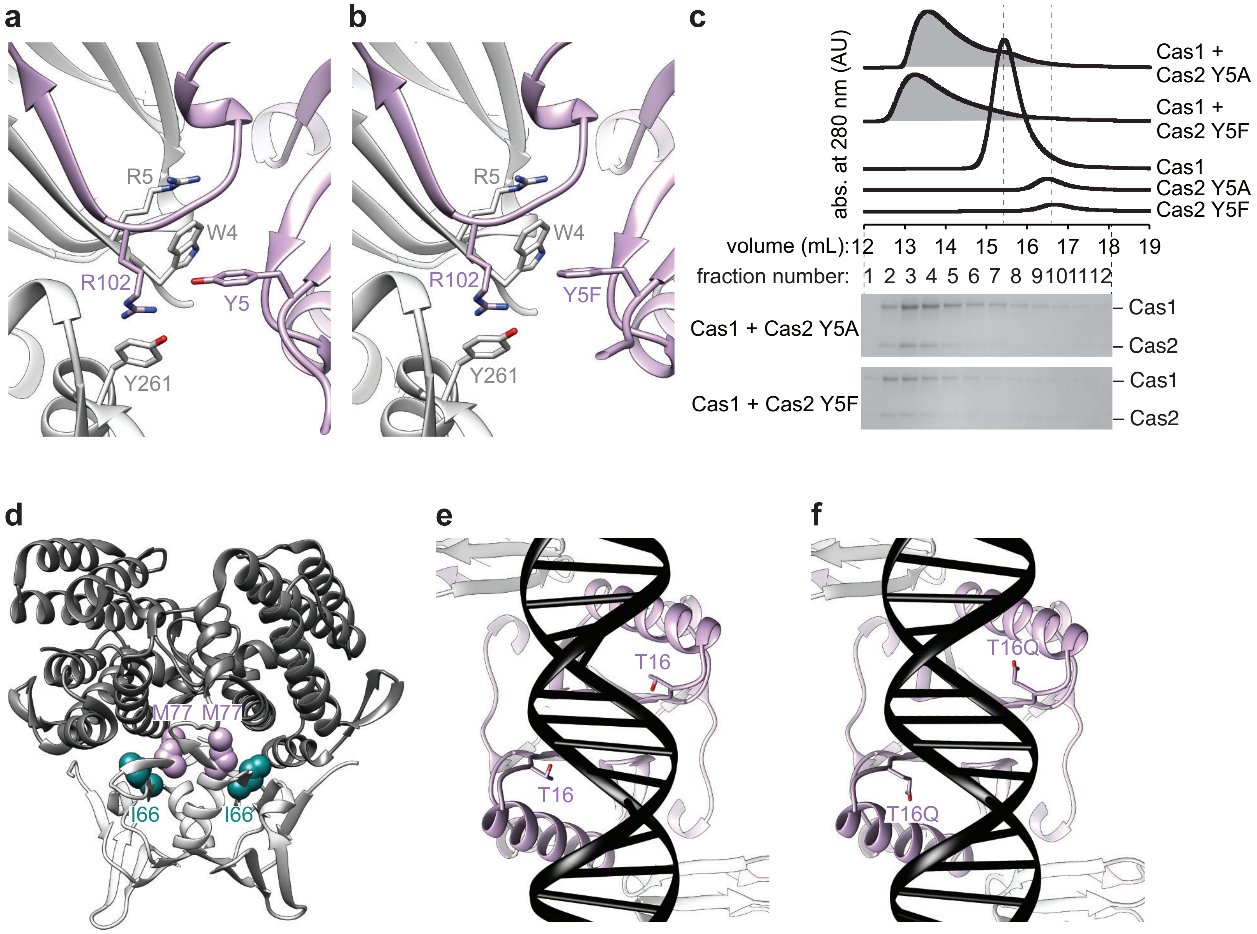


**Supplementary Fig. 18 | Structural context of residue changes that enhance spacer acquisition. a**, **b** Structural context of Cas2 Y5 (**a**) and Y5F (**b**) in AlphaFold3 models. Cas1 is shown in grey and Cas2 in purple. **c** Interaction of Cas1 (2 equiv.) and indicated Cas2 variants as determined by analytical SEC (top), and Coomassie blue SDS-PAGE analysis of corresponding elution fractions (bottom). Elution profiles of individual protein standards are shown. **d** Structural context of Cas1 M77 and I66 in the *S. pyogenes* Cas1 dimer (PDB 4ZKJ), with the N-terminal domains and linkers (residues 1–87) shown in light grey, and the C-terminal α-helical domains (residues 88–289) in dark grey. **e**, **f** Structural context of Cas2 T16 (**e**) and T16Q (**f**) in AlphaFold3 models. Cas1 is shown in grey, Cas2 in purple, and the DNA prespacer in black.
