# Supplementary material for: Deep mutational scanning identifies Cas1 and Cas2 variants that enhance type II-A CRISPR-Cas spacer acquisition
Source: Nat Commun. 2025 Jul 1;16:5730. doi: 10.1038/s41467-025-60925-9 (PMC12216489; doi:10.1038/s41467-025-60925-9)
Supplement: Supplementary file 2 — Description of Additional Supplementary Files [file 41467_2025_60925_MOESM2_ESM.pdf]

Description of Additional Supplementary Files

File Name: Supplementary Data 1
Description: Amino acid preferences, differential selection and functional scores of residue substitutions obtained from deep mutational scanning of *Streptococcus pyogenes* Cas1, Cas2 and Csn2.

File Name: Supplementary Data 2
Description: Plasmids used in this study.

File Name: Supplementary Data 3
Description: Oligonucleotide primers used in this study.
